# ^68^Ga Extemporaneous Preparations in Radiopharmacy

**DOI:** 10.3390/pharmaceutics17070802

**Published:** 2025-06-20

**Authors:** Marzia Rizzello, Anna Pacelli, Maria Domenica De Bari, Annalisa Cutrignelli, Rosa Maria Iacobazzi, Antonio Lopalco, Nunzio Denora

**Affiliations:** 1 BeForPharma S.r.l., Volga Street c/o Fiera del Levante Pad. 129, 70132 Bari, Italy; m.rizzello@beforpharma.com (M.R.); a.pacelli@beforpharma.com (A.P.); m.debari@beforpharma.com (M.D.D.B.); 2 Department of Pharmacy-Pharmaceutical Sciences, University of Bari Aldo Moro, 4, E. Orabona Street, 70125 Bari, Italy; annalisa.cutrignelli@uniba.it (A.C.); rosamaria.iacobazzi@uniba.it (R.M.I.); nunzio.denora@uniba.it (N.D.)

**Keywords:** gallium-68, PSMA, DOTATOC, FAPI, extemporaneous preparations, radiopharmaceuticals, nuclear medicine, NBP, GMP

## Abstract

Gallium-68 (^68^Ga) radiopharmaceuticals are increasingly used in nuclear medicine due to their rapid production capabilities and exceptional specificity in molecular imaging applications. Several of these tracers have demonstrated remarkable clinical efficacy across various oncological conditions, including prostate cancer, neuro-endocrine tumours, and cancers expressing fibroblast activation protein. Commercial kits allow the use of the standardised production protocol, but extemporaneous preparations are the economic and flexible alternatives, particularly within hospital-based radiopharmacy settings. However, such preparations need meticulous conformity to quality control measures and regulation to ensure safety and effectiveness. This review provides an analysis of current methodologies employed in ^68^Ga extemporaneous preparations and examines pertinent regulatory frameworks. Further clinical validation trials and technical advancement remain essential to facilitate the routine clinical practice’s widespread usage and long-term feasibility of such preparations.

## 1. Introduction

Nuclear medicine has transformed modern healthcare by significantly enhancing disease diagnosis and treatment methodologies. Central to this progress is the implementation of radiopharmaceuticals: compounds that integrate radioactive isotopes with biologically active molecules to target specific physiological or pathological processes. Among the diverse radionuclides employed in nuclear medicine, gallium-68 (^68^Ga) is particularly noteworthy for its distinctive properties. With a brief half-life of approximately 68 min and positron emission capabilities, ^68^Ga demonstrates exceptional efficacy for positron emission tomography (PET) imaging applications [1]; furthermore, the overall versatility of this radionuclide is reinforced by a supply chain that includes multiple readily available sources, simple radiochemical handling, and several routes for GMP-compliant radiopharmaceutical production. This unique combination of features is unmatched by any other PET radionuclide [2].

**Figure 1:**
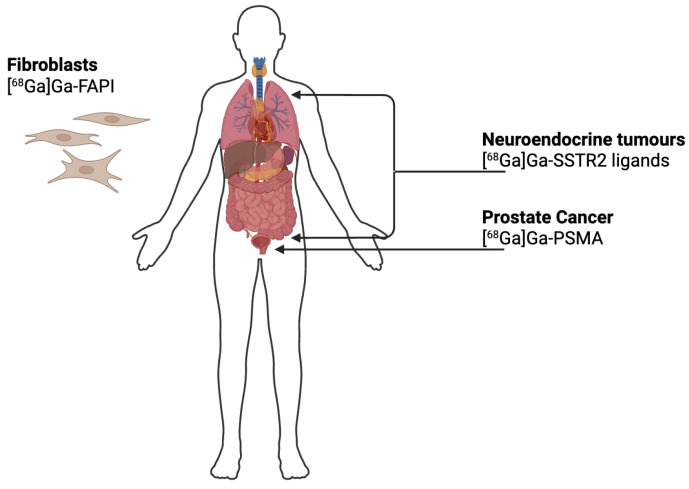
Schematic overview of the principal molecular targets of ^68^Ga-labelled radiopharmaceuticals used in clinical imaging: [^68^Ga]Ga-PSMA targets prostate-specific membrane antigen used in the diagnosis and staging of prostate cancer; [^68^Ga]Ga-SSTR2 ligands bind to somatostatin receptors expressed by neuroendocrine tumours; [^68^Ga]Ga-FAPI targets fibroblast activation protein (FAP) overexpressed in cancer-associated fibroblasts, allowing visualisation of the tumour stroma in a variety of malignancies.

In recent years, ^68^Ga applications have expanded significantly, especially in prostate cancer and neuroendocrine tumour imaging. Some of the most commonly used ^68^Ga radiopharmaceuticals include prostate-specific membrane antigen (PSMA) [3] ligands and somatostatin receptor (SSTR2)-targeting tracers like DOTA-TOC [4]. More recently, fibroblast activation protein inhibitor (FAPI) tracers have emerged as promising agents for diagnosing a broader spectrum of malignancies beyond prostate and neuroendocrine tumours [5]. Beyond these established radiopharmaceuticals, it is noteworthy that numerous novel ^68^Ga-labelled tracers are currently under investigation. These include [^68^Ga]Ga-NeoB, a DOTA-coupled gastrin releasing peptide receptor (GRPR) antagonist with high affinity for GRPR and demonstrated good in vivo stability [6]. Additionally, [^68^Ga]Ga-PentixaFor, a PET agent targeting CXCR4, is emerging as a versatile radiotracer with promising applications across oncology, cardiology, and inflammatory diseases [7]. Furthermore, αvβ6-integrin has emerged as a promising molecular target for both oncological and non-oncological conditions [8], expanding the potential scope of ^68^Ga-based imaging agents, such as [^68^Ga]Ga-Trivehexin. These compounds (Figure 1) have transformed tumour detection, allowing for precise localisation, earlier diagnosis, and better treatment planning. Indeed, in the field of oncology, the early detection of small lesions remains a critical factor. Compared to conventional imaging modalities, PET radiotracers, particularly those labelled with ^68^Ga, offer the distinct advantage of noninvasively mapping and quantifying molecular and biochemical processes in vivo. These tracers distribute systemically, enabling high-resolution, three-dimensional visualisation of specific biological targets, and allow for the rapid acquisition of dynamic tomographic data, facilitating efficient whole-body assessments. This functional imaging capability significantly exceeds the purely anatomical information provided by techniques such as CT or MRI [2].

Given the short physical half-life of ^68^Ga-radiopharmaceuticals, small-scale production at non-industrial facilities, such as hospital-based radiopharmacies, is often essential. Simultaneously, there is an increasing demand for personalised or precision medicine, with treatments specifically tailored to the individual clinical needs of each patient [9].

This review delineates the current landscape of extemporaneous ^68^Ga-labelled radiopharmaceutical preparation, encompassing the radiochemical characteristics, production methodologies, clinical applications, and regulatory frameworks governing their use. By integrating technical insights with regulatory considerations, it addresses the unique challenges inherent to small-scale, hospital-based manufacturing, emphasising the critical importance of standardised procedures and stringent quality control.

## 2. ^68^Ga in Nuclear Medicine

### 2.1. Chemical and Physical Properties of ^68^Ga

Out of the 40 identified Ga radioisotopes, three—^66^Ga, ^67^Ga, and ^68^Ga—have found applications in nuclear medicine. Among these, ^68^Ga is particularly valuable due to its positron emission properties, making it an ideal candidate for molecular imaging using PET [10], as summarised in Table 1. In contrast, ^67^Ga, most commonly administered as ^67^Ga-citrate, has been widely used for infection and inflammation imaging. ^66^Ga has also been explored for similar diagnostic purposes and has been employed as an alternative to ^68^Ga in PET imaging of radioligands with slower pharmacokinetics [11]. However, its clinical use remains limited due to its relatively short half-life and higher positron energy, which may compromise image resolution and dosimetry profiles [12].

Indeed, ^68^Ga is a positron-emitting radionuclide with a physical half-life of 67.7 min. It decays predominantly by β⁺ emission (≈89%), with a maximum positron energy (E_max_) of 1899 keV and an average positron energy of approximately 770 keV. The emitted positrons undergo annihilation with electrons, resulting in the production of two 511 keV photons that are detected by PET scanners [1]. One of the main advantages of ^68^Ga is this short physical half-life, which is compatible with the pharmacokinetics and biological half-lives of numerous radiopharmaceuticals, including peptides, oligonucleotides, and affibody molecules [8]. ^68^Ga rapid decay kinetics enable efficient imaging within a clinically relevant timeframe. Although such a short half-life might be perceived as a limitation, it offers important clinical benefits by allowing adequate time for radiolabelling, quality control, and image acquisition, while minimising radiation exposure to patients [9]. Moreover, the [^68^Ga]Ga^3+^ cation can form stable complexes with a wide range of ligands containing donor atoms such as oxygen and nitrogen [1], further expanding its applicability in molecular imaging.

### 2.2. Production of ^68^Ga

**Figure 2:**
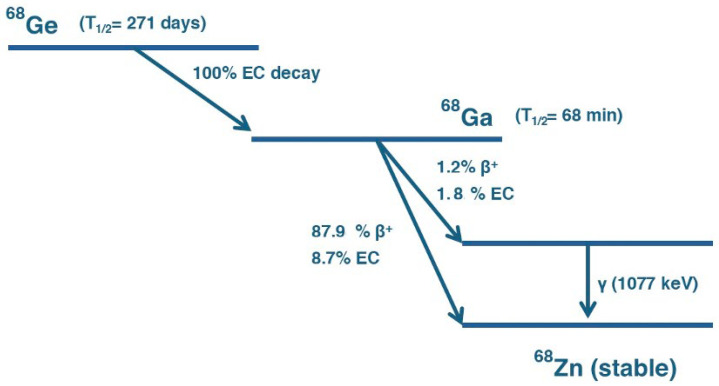
Decay scheme of ^68^Ge into ^68^Ga. ^68^Ge decays via EC with a half-life of 271 days to ^68^Ga. ^68^Ga subsequently decays with a half-life of 68 min through multiple pathways: predominantly via positron emission (β⁺, 87.9%) and electron capture (EC, 8.7%) to the ground state of stable Zinc-68 (^68^Zn), and to a lesser extent via β⁺ (1.2%) and EC (1.8%) to an excited state of ^68^Zn, followed by gamma emission (γ, 1077 keV).

The main source of ^68^Ga in many nuclear medicine radiopharmaceuticals is the ^68^Ge/^68^Ga generator. This device relies on the principle of radioactive decay coupled with chemical separation, enabling the continuous on-site availability of ^68^Ga derived from its long-lived parent isotope, ^68^Ge. In this system, ^68^Ge is immobilised on a solid chromatographic matrix and undergoes electronic capture (EC) decay to form ^68^Ga, as illustrated in Figure 2.

**Figure 3:**
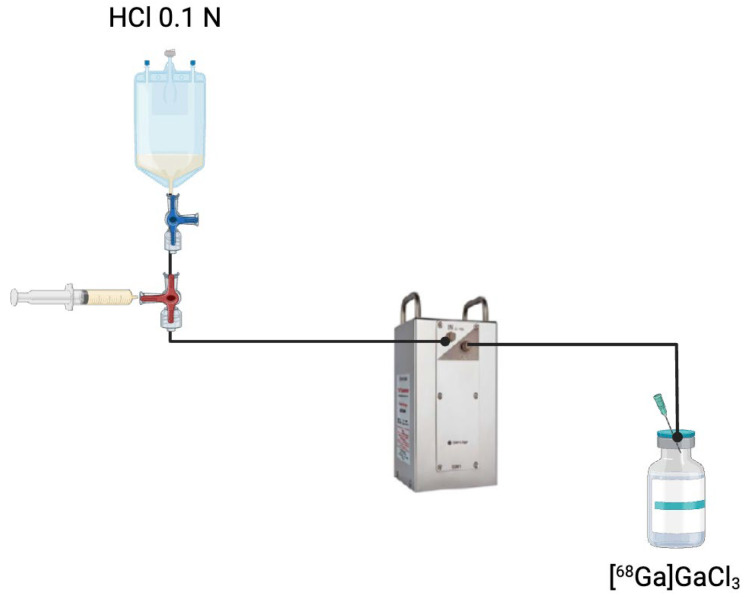
Elution process of a ^68^Ge/^68^Ga generator. A solution of HCl 0.1 N is passed through the generator column containing immobilised ^68^Ge to extract [^68^Ga]GaCl_3_. This eluate is collected and subsequently used for radiopharmaceutical applications.

Since ^68^Ge has a half-life of approximately 271 days, it continuously generates ^68^Ga over an extended period. The ^68^Ga produced remains trapped in the generator until it is extracted through an elution process [13]. To collect ^68^Ga, a small volume of HCl solution is passed through the generator column, selectively dissolving the accumulated ^68^Ga while leaving the parent ^68^Ge behind. The resulting solution, which contains [^68^Ga]GaCl_3_, can then be used directly for radiopharmaceutical preparations (Figure 3). After each elution, the generator begins accumulating ^68^Ga again; within approximately 68 min (one half-life of ^68^Ga), about 50% of the total radioactivity is restored, and 4 h later, it is over 91% [14], allowing multiple dose extractions per day. Elution conditions vary according to the generator’s resin composition and radiochemical purity specifications. For example, the GalliaPharm^®^ generator (Eckert & Ziegler Radiopharma, GmbH, Berlin, Germany). is typically eluted with 10 mL of 0.1 M HCl over 5 min [15]. The IRE ELiT generator (IRE ELiT S.A., Fleurus, Belgium) employs 7 mL of 0.1 M HCl at a controlled flow rate of 1 mL/min [16], while the GalliAd^®^ generator (IRE ELiT S.A., Fleurus, Belgium). is eluted with 1.1 mL of 0.05 M HCl over a minimum of 3 min [16].

Metal impurities commonly present in the ^68^Ga generator eluate can negatively impact the radiolabelling efficiency of bifunctional chelator-derived radiopharmaceuticals by competing with gallium ions during complexation. These impurities may lead to reduced yield, lower radiochemical purity, and potential instability of the final product [17]. To mitigate these effects, it is essential to implement strict measures during reagent preparation and handling. Contact with metallic surfaces should be avoided throughout the process, and the use of plastic-based disposable materials is strongly recommended. Additionally, reagents should be prepared using trace-metal grade chemicals and ultrapure deionised water with minimal metal content. Standard laboratory glassware is discouraged, as it may introduce contaminating ions or adsorb gallium. In some radiopharmacy settings, working areas such as hot cells or fume hoods are lined with adhesive plastic films to minimise corrosion and prevent iron-based contamination, further preserving the quality of the radiolabelling process [18].

Over the years, different types of ^68^Ge/^68^Ga generators have been developed, utilising various solid-phase supports such as titanium dioxide, tin dioxide, silica, nano-zirconia, and modified polymer-based materials; these supports retain the long-lived parent nuclide ^68^Ge within the column and enable the elution of ^68^Ga in a chemical form suitable for direct radiolabelling [19]. The ^68^Ge/^68^Ga generator has several advantages for medical centres that are located far from ^68^Ga distribution facilities. Generators do not require specialised infrastructure, high energy consumption, or highly trained personnel for operation and maintenance, making them an attractive choice for many nuclear medicine centres [14]. Additionally, when compared to other PET radionuclides such as fluorine-18 (^18^F), ^68^Ga generators provide greater operational autonomy and simplicity. The production of ^18^F, indeed, depends on a cyclotron and involves more complex radiochemical synthesis, which often confines its use to centralised radiopharmacies or necessitates a well-established distribution system [20]. The technical characteristics of commercially available ^68^Ge/^68^Ga generators, including elution yield, initial ^68^Ge activity, ^68^Ge breakthrough, and operational differences such as the use of built-in versus external eluents, have been comprehensively reviewed in other publications [14,19,21]. To avoid redundancy, these comparative aspects are not discussed in detail in this review.

In recent years, researchers have been exploring cyclotron-based production as an alternative strategy to diversify the supply of ^68^Ga beyond generator-based systems and to provide higher activity levels. This approach is based on the ^68^Zn (p, n) ^68^Ga nuclear reaction and can be implemented using either solid or liquid targets [22]. In the solid target method, enriched ^68^Zn is employed in different forms, such as electroplated, pressed, foil, or molten targets. The process involves proton irradiation of the solid target using medical cyclotrons [23]. The target is then dissolved using either concentrated HCl or HNO_3_. The separation and purification of ^68^Ga are achieved through one-, two-, or three-column chromatography techniques, maximising the apparent molar activity (AAm) and ensuring high-purity ^68^Ga suitable for radiopharmaceutical applications [24]. Studies using the GE PETtrace cyclotron have shown promising results, with end-of-purification (EOP) activity exceeding 3.7 GBq, and could be distributed to other nuclear medicine centres [25]. The highest end-of-bombardment (EOB) activity reported was over 370 GBq [24].

Recent advances have led to the development of liquid target production of ^68^Ga, which offers an alternative for facilities without solid target infrastructure. Instead of irradiating a solid material, a liquid solution of enriched [^68^Zn]Zn(NO_3_)_2_ in HNO_3_ is used as the target medium [26]. This approach minimises target preparation time and simplifies the process by eliminating the need for complex target dissolution steps. Liquid target production has demonstrated promising results, with studies reporting EOB activities exceeding 9 GBq when using optimised concentrations of Zn(NO_3_)_2_ and HNO_3_ [27]. To achieve high-purity ^68^Ga, the irradiated solution undergoes a two-column purification process, typically using Zr resin and TK200 resin. After separation, the final ^68^Ga is formulated as [^68^Ga]GaCl_3_, which can then be used for radiosynthesis [24].

### 2.3. ^68^Ga Chelators

**Figure 4:**
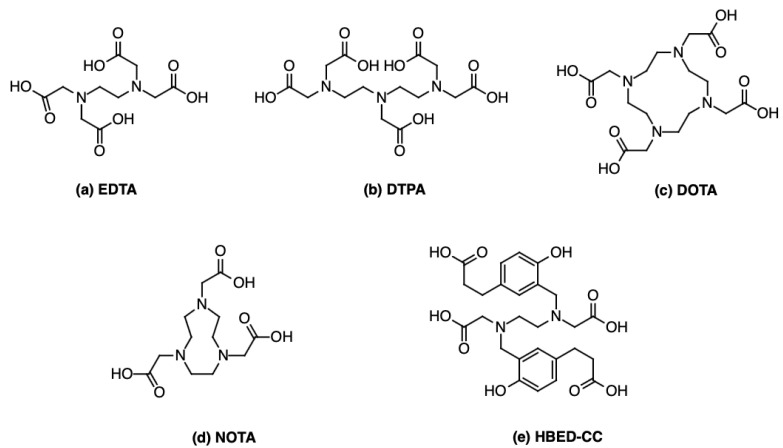
Chemical structure of several ^68^Ga chelators: (**a**) EDTA; (**b**) DTPA; (**c**) DOTA; (**d**) NOTA; (**e**) HBED-CC.

Chelators play a fundamental role in the development of ^68^Ga radiopharmaceuticals. Ga^3+^ is a hard Lewis acid that preferentially forms coordination complexes with oxygen, nitrogen, and sulphur donor atoms. The most stable ^68^Ga complexes adopt an octahedral geometry, meaning that six donor atoms are ideally involved in the chelation process. Functional groups commonly used for Ga^3+^ coordination include amines, carboxylates, hydroxamates, and phenolates [28]. Choosing the right chelating agent is essential to ensure that ^68^Ga remains stably bound to the radiopharmaceutical throughout imaging. Chelators must exhibit strong thermodynamic stability, fast complexation kinetics, and minimal dissociation under physiological conditions [29]. The first clinically relevant ^68^Ga complexes were based on EDTA (ethylenediaminetetraacetic acid) (Figure 4a). In the early days of ^68^Ga imaging, EDTA was used to form ^68^Ga complexes directly in generator eluates, which were then administered for perfusion imaging [30]. However, EDTA chelates exhibited poor in vivo stability, leading to transchelation with endogenous metal ions such as Fe^3+^ and Zn^2+^, reducing their clinical applicability [31]. Building upon EDTA, DTPA (diethylenetriaminepentaacetic acid, Figure 4b) emerged as a more effective chelator for ^68^Ga. DTPA derivatives have been widely used in radiopharmaceutical chemistry for various radioisotopes, but when applied to ^68^Ga, they still demonstrated moderate in vivo stability concerns [30]. Their acyclic nature makes them more prone to metal exchange and dissociation in physiological conditions.

To overcome these limitations, macrocyclic chelators were introduced. Some of the most widely used macrocyclic chelators include the following:

- DOTA (1,4,7,10-Tetraazacyclododecane-1,4,7,10-tetraacetic acid, Figure 4c), which forms exceptionally stable ^68^Ga complexes in vivo, preventing dissociation or transmetallation under physiological conditions [32]. For instance, the study performed by Oehlke and colleagues analysed the stability of [^68^Ga]Ga-DOTATATE towards transmetallation with Cu^2+^, Fe^3+^, Pb^2+^, and Zn^2+^, and found that only the addition of Cu^2+^ at 95 °C leads to noticeable transmetallation [33].
  DOTA radiolabelling requires high temperatures (~90 °C) for approximately 10 min. This heating step is crucial, due the rigidity of its macrocyclic structure, which must undergo conformational rearrangement to accommodate the Ga^3+^ ion within its coordination cavity [30]. The ideal pH range for DOTA chelation is 3.5–4.0. At lower pH, DOTA becomes overly protonated, while at higher pH, Ga^3+^ may form insoluble species [34]. Structurally, DOTA provides an octadentate N_4_O_4_ coordination environment and a relatively large macrocyclic cavity that may lead to slower complexation kinetics and reduced resistance to transchelation due to imperfect fitting with the ionic radius of Ga^3+^ [35].
- NOTA (1,4,7-Triazacyclononane-1,4,7-triacetic acid), which forms highly stable complexes at room temperature [30]. This property is attributed to the intrinsic structural features of NOTA (Figure 4d): its compact macrocyclic geometry, hexadentate N_3_O_3_ coordination, and smaller cavity size, which better matches the ionic radius of Ga^3+^, facilitating faster complex formation and superior kinetic stability [36]. Ga^3+^-NOTA complexes exhibit an exceptionally high stability constant (logK = 30.98), significantly higher than that of Ga³⁺-DOTA (logK = 21.33), highlighting NOTA’s stronger affinity for Ga^3+^ and more favourable thermodynamics [32]. Structurally, NOTA forms slightly distorted octahedral complexes with facially arranged donors, allowing the formation of multiple five-membered chelate rings with minimal strain, unlike DOTA. This lower energetic barrier enables efficient complexation even at mild conditions [37]. In terms of in vivo stability, although NOTA forms more inert complexes with Ga^3+^ than DOTA, the overall biological performance of the radiopharmaceutical also depends on the charge, hydrophilicity, and pharmacokinetics of the resulting bioconjugate. For example, DOTA-based vectors may display more favourable biodistribution in certain contexts, despite their lower thermodynamic stability [32]. Similarly to DOTA, NOTA has shown strong resistance to transmetallation, particularly against biologically relevant cations such as Zn^2+^ and Ca^2+^; additionally [33]. NOTA, provides enhanced kinetic inertness even under physiological conditions [32,38].

Meanwhile, efforts have continued to refine non-macrocyclic chelators that offer faster labelling kinetics at room temperature. The most notable example is HBED-CC (N,N′-bis(2-hydroxy-5-(carboxyethyl)benzyl)ethylenediamine-N,N′-diacetic acid, Figure 4e), an acyclic hexadentate ligand that coordinates Ga^3+^ through phenolic, amine, and carboxylate donor atoms to form stable complexes. Despite lacking a macrocyclic structure, HBED-CC achieves high thermodynamic stability and kinetic inertness due to the optimal spatial arrangement and strong coordinating ability of its donor groups, allowing it to efficiently chelate ^68^Ga at room temperature and at pH ~4 [39].

### 2.4. Conditions of ^68^Ga Complexation

The production of ^68^Ga-labelled radiopharmaceuticals requires rigorous control over reaction conditions to ensure efficient complexation and full compliance with regulatory standards. This is particularly crucial given the short half-life of the radionuclide, which imposes significant time constraints on the production process.

In aqueous solutions, Ga^3+^ ions exhibit acid–base behaviour that results in the formation of multiple hydrolysed species [39]. At pH values below 3, Ga predominantly exists as the fully hydrated complex [Ga(H_2_O)_6_]^3+^. As the pH increases, progressive deprotonation occurs, leading to the formation of hydroxo complexes such as [Ga(OH)]^2+^, [Ga(OH)_2_]^+^, [Ga(OH)]_3_, and [Ga(OH)_4_]^−^. The speciation is also temperature dependent. At 25 °C, [Ga(OH)]^2+^ is the main species between pH 3 and 5, whereas at 100 °C, [Ga(OH)_2_]^+^ predominates. Above pH 5, [Ga(OH)_4_]^−^ becomes the dominant form, regardless of temperature. These equilibria highlight the importance of maintaining an appropriate pH to ensure optimal Ga availability for complexation [40].

The efficiency of the labelling process is also critically dependent on the choice of chelating agent [41]. An ideal chelator should form a stable complex with the radiometal under mild conditions and at very low (nano- to micromolar) concentrations of the radiometal. It should also be capable of quantitatively binding the radionuclide even when used in low molar amounts, ensuring high molar activity of the final product. This is essential to prevent competition between the unlabelled precursor and the radiolabelled molecule for target receptors in vivo, which could compromise the specificity of the radiopharmaceutical. Furthermore, the complex must be sufficiently stable under physiological conditions to avoid premature release of the radiometal, which would result in undesirable accumulation in non-target tissues [41].

Another critical component of the synthesis is the selection of an appropriate buffer system [42]. Buffers are selected based on their pKa and ability to maintain the pH between 3.5 and 5.0, the optimal range for Ga complexation. Clinically suitable buffers must be pharmaceutically approved and not form themselves strong complexes with Ga, thus interfering with the labelling. For instance, citrate binds Ga^3+^ strongly and is unsuitable as a buffer for ^68^Ga radiolabelling, as proved by its clinical use in inflammation imaging [43]. Alternatives like HEPES, succinate, glutamate, and oxalate offer good pH control and can enhance labelling yields [42], but many lack pharmacopoeial approval. HEPES improves labelling efficiency by reducing precursor mass requirements. However, its use is limited by the European Pharmacopoeia’s strict 200 μg per dose limit for intravenous administration [44], based on limited toxicity data. Yet, studies have shown that much higher doses are well tolerated [45], and HEPES is found in approved drugs, indicating its safety at levels exceeding those used in radiopharmacy. These findings suggest that a reassessment of current regulatory limits may be warranted, potentially expanding the clinical applicability of HEPES-buffered ^68^Ga radiopharmaceuticals without compromising patient safety [46].

## 3. Regulatory Guidelines of ^68^Ga Radiopharmaceuticals

### 3.1. Regulatory Guidelines and Radiation Protection Regulations

In the field of radiopharmaceutical production, a distinction must be made among the following:

- Industrial-scale ready-to-use radiopharmaceuticals, produced under full GMP and subject to centralised marketing authorisation procedures;
- Radiopharmaceutical precursors, manufactured under GMP and intended for subsequent radiolabelling in healthcare facilities;
- Small-scale extemporaneous radiopharmaceutical preparations, compounded within hospital or academic radiopharmacy settings under national exemptions and pharmacopoeial standards [47].

This review primarily focuses on the third category, which includes magistral and officinal preparations of ^68^Ga-labelled radiopharmaceuticals produced onsite in nuclear medicine departments, often under time-sensitive and patient-specific conditions.

The regulatory framework for radiopharmaceuticals in the European Union was first introduced with Directive 89/343/EEC, which primarily focused on radiation protection measures and compliance with pharmacopoeial standards [48]. Since 1989, radiopharmaceuticals have been formally recognised as medicinal products and their current legal basis is established by Directive 2001/83/EC, the Community Code relating to medicinal products for human use [49]. This Directive provides exemptions from the requirement for marketing authorisation (MA) under Articles 3 and 5(1), which allow the use of magistral and officinal formulas, as well as preparations authorised under national provisions, including extemporaneously radiopharmaceutical preparations [49]. This exemption plays a key role for radiopharmaceuticals, as small-scale preparations at non-industrial sites, such as nuclear medicine departments, are often necessary due to the short physical half-life of many radionuclides, including ^68^Ga [50]. Extemporaneously radiopharmaceutical preparations are commonly prepared on-site, often as single-dose formulations customised to specific clinical needs. They typically include magistral preparations, compounded in a pharmacy in accordance with an individual medical prescription, and officinal preparations, produced in line with a pharmacopoeial monograph. In both cases, the radiopharmaceuticals are intended for direct administration to patients [51]. However, the evolution of nuclear medicine practices and the increasing complexity of radiopharmaceutical production have highlighted critical limitations in the current regulatory framework. As outlined by the European Association of Nuclear Medicine (EANM), Directive 2001/83/EC remains largely focused on traditional kit-based preparations and fails to adequately address the regulatory needs of novel, in-house, and complex radiopharmaceutical formulations. This regulatory gap results in inconsistencies across Member States, disproportionate administrative burdens, and potential barriers to innovation and patient access [52].

In Italy, Directive 2001/83/EC has been transposed into national law through Legislative Decree No. 219 of 24 April 2006 [53]. Within this framework, the preparation of magistral and officinal radiopharmaceuticals is permitted under the exemption provided by Article 3 of the Directive. The quality requirements for magistral formulations are defined in the “Norme di buona preparazione in medicina nucleare” (Good compounding practice in nuclear medicine, NBP-MN) published in the Official Pharmacopeia of the Italian Republic [54]. In parallel, radioprotection aspects related to the extemporaneous preparation and clinical use of radiopharmaceuticals are regulated by Legislative Decree No. 101 of 31 July 2020 [55], which transposes the European Directive 2013/59/Euratom [56] into national law. This decree establishes the basic safety standards for protection against the risks associated with exposure to ionising radiation, covering all activities involving radioactive substances, including those performed within nuclear medicine departments. Among its provisions, the decree assigns key responsibilities to the Radiation Protection Expert (Esperto di Radioprotezione, ERP), who must ensure that shielding, handling protocols, and monitoring systems are appropriate to minimise occupational and environmental exposure [57]. For small-scale preparations, the legislation requires dedicated infrastructure, including containment systems (e.g., hot cells), dose calibrators, contamination control measures, and personal dosimetry devices [57].

### 3.2. European Pharmacopoeia

The European Pharmacopoeia (Eur. Ph.) establishes official standards for the qualitative and quantitative composition of medicinal products, as well as for the analytical procedures applicable to both finished pharmaceuticals and raw materials used in their manufacture [58]. In the field of radiopharmaceutical production, the General Monograph 0125 “Radiopharmaceutical Preparations” [59], provides a comprehensive regulatory framework outlining the fundamental quality requirements applicable to all radiopharmaceuticals. In addition to this general guidance, the Eur. Ph. includes approximately 70 individual monographs covering radiolabelled preparations and radioactive precursors. Among these, specific monographs have been established for [^68^Ga]Ga-PSMA-11 (Monograph 3109) [60] and [^68^Ga]Ga-DOTATOC, also known as edotreotide (Monograph 3098) [61], which define the quality specifications for the identity, content, purity, and radiochemical characteristics of these extemporaneous radiopharmaceuticals. These monographs are distinct from those intended for ready-to-use industrial radiopharmaceuticals and reflect the specific constraints and operational realities of extemporaneous preparation in hospital settings. In particular, they often allow for adapted quality testing strategies due to the short half-life and immediate-use nature of ^68^Ga-labelled products.

In the case of commercially available kits, the Eur. Ph. allows partial exemptions from certain tests, provided that the preparation is compliant with a marketing authorisation and the generator specified in the approved documentation is used. However, if a licensed kit is used outside of its intended specifications, or with a non-authorised generator, a full analytical release profile is required [62].

### 3.3. Good Manufacturing Practice Guidelines

Good Manufacturing Practice (GMP) refers to a set of internationally recognised standards that ensure the consistent production and control of medicinal products according to quality standards appropriate for their intended use [63]. In the European Union, GMP principles are codified in EudraLex Volume 4, which is structured into three main parts:

- Part I, Basic Requirements for Medicinal Products, outlines the GMP standards applicable to the manufacture and control of finished pharmaceutical products for both human and veterinary use. It is organised into nine chapters: Chapter 1 defines the Pharmaceutical Quality System necessary to consistently produce medicines that meet regulatory and patient expectations; Chapter 2 specifies the responsibilities, qualifications, and training requirements for personnel involved in GMP activities; Chapter 3 outlines the design, maintenance, and control standards for premises and equipment; Chapter 4 addresses documentation management, including procedures, records, and batch documentation; Chapter 5 describes principles for production operations, contamination control, and process validation; Chapter 6 establishes the requirements for sampling, testing, release, and stability studies; Chapter 7 regulates the management of outsourced activities; Chapter 8 defines procedures for handling complaints, investigating quality defects, and executing product recalls; and Chapter 9 covers the requirements for self-inspections to ensure ongoing GMP compliance.
- Part II, Basic Requirements for Active Substances used as Starting Materials, defines the GMP framework for the production of active pharmaceutical ingredients (APIs), focusing on quality, purity, and traceability throughout the manufacturing process.
- Part III, GMP-related Documents, compiles complementary guidelines, such as ICH Q9 on Quality Risk Management and ICH Q10 on Pharmaceutical Quality Systems, which extend and reinforce the broader quality framework necessary for maintaining GMP compliance.

GMP principles are not fully applicable to extemporaneously prepared radiopharmaceuticals, due to their intrinsic characteristics such as short physical half-lives, the need for aseptic handling, and time-critical preparation. To address these specific challenges and ensure consistent quality and regulatory compliance, targeted technical annexes have been developed to guide production practices across all stages [50].

Annex 1 of EudraLex Volume 4 (“Manufacture of Sterile Medicinal Products”), focused on sterile manufacturing, is particularly applicable to radiopharmacy [64]. It provides detailed guidance on environmental classifications (Grades A to D), cleanroom standards, personnel training, and environmental monitoring, all essential for maintaining aseptic conditions during critical steps like sterile filtration and final dose preparation [64]. The 2023 revision of Annex 1 of EudraLex Volume 4 introduced significant changes compared to the previous version, reflecting the evolving regulatory expectations for sterile manufacturing. A key advancement is the formal incorporation of a contamination control strategy (CCS), which requires manufacturers to establish a holistic, documented, and proactive approach to contamination risk assessment and mitigation across the entire lifecycle of the product. Unlike the previous version, which provided more prescriptive guidance focused on individual aspects of sterility assurance, the current Annex emphasises an integrated quality-by-design philosophy, risk-based decision-making, and continuous process verification. Greater attention is also given to topics such as environmental monitoring, personnel gowning qualification, and the use of barrier technologies (e.g., Restricted Access Barrier Systems and isolators) to minimise contamination risks.

Annex 3 of EudraLex Volume 4 (“Manufacture of Radiopharmaceuticals”) specifically provides recommendations for the preparation of radiopharmaceuticals, including those prepared extemporaneously [65]. It serves as a key regulatory reference for aligning hospital-based radiopharmaceutical preparation with the broader principles of pharmaceutical quality and patient safety. The Annex mandates compliance with GMP Parts I and II and emphasises the importance of a robust quality assurance system, including contamination control, aseptic production practices, and specialised training in both GMP and radiation protection. It also accommodates the unique operational constraints of radiopharmaceuticals by allowing specific concessions to standard GMP practices such as permitting most container labelling to occur prior to manufacturing to reduce radiation exposure and by authorising conditional batch release when full quality control results are not available before administration. For radiopharmaceuticals with short half-lives such as ^68^Ga-labelled preparations, microbiological testing (e.g., sterility) is typically completed only after the product has already been administered to the patient. Therefore, Annex 3 explicitly permits the release of the product prior to the availability of sterility test results, provided that all other critical quality attributes (e.g., radiochemical purity, pH, radionuclidic identity, endotoxins) are compliant and that a validated aseptic process is in place. Furthermore, Annex 3 incorporates additional GMP elements by referring to Annex 11 for computerised systems and Annex 13 for investigational medicinal products. Annex 3, therefore, introduces a more rigorous and harmonised framework, bringing radiopharmaceutical manufacturing closer to mainstream pharmaceutical GMP standards while still recognising the specific needs of nuclear medicine.

Across the European Union, however, the degree of GMP application in radiopharmaceutical preparation varies by Member State. In countries such as France and Italy, a tailored approach known as Good Compounding Practice has been adopted, adapting GMP principles to the specific context of hospital-based preparation. In contrast, Member States including Belgium, Austria, and Finland apply the standards defined in PIC/S Annex 3, which is specifically designed for the preparation of medicinal products in healthcare establishments. In other jurisdictions, such as Germany, Spain, Denmark, and the United Kingdom, compliance with the full scope of GMP requirements is mandatory, regardless of the production scale or setting. A detailed review on how different European countries approach this topic can be found on [66].

### 3.4. International Council for Harmonisation Guidelines

The validation of analytical procedures in pharmaceutical development is regulated by the International Council for Harmonisation (ICH) guidelines, notably, ICH Q2 (R2) [67]. Although this framework is widely applied to conventional pharmaceutical products, its direct implementation in the context of extemporaneous radiopharmaceutical preparations raises several challenges due to the distinctive nature of these compounds.

Radiopharmaceuticals, particularly those involving short-lived radionuclides, as ^68^Ga, are characterised by rapid radioactive decay and the frequent unavailability of isolated reference standards for radioactive components [68]. These peculiarities are not fully addressed by the ICH Q2 (R2) guidelines. Furthermore, the radioactive drug substance is often inseparable from the formulation matrix, necessitating either significant adaptations to conventional validation protocols or the implementation of alternative strategies. As a result, although ICH Q2 (R2) offers a valuable framework, it must be complemented by radiopharmacy-specific methodologies [69].

Recognising these limitations, the European Association of Nuclear Medicine (EANM) has issued specialised guidance tailored to the validation of analytical methods for radiopharmaceuticals [69]. These documents incorporate parameters critical to radiopharmaceutical quality, including radionuclidic identity and radiochemical purity, and promoted their formal consideration as exceptions to conventional ICH procedures [47].

In alignment with ICH Q2 (R2), the validation of analytical methods for extemporaneously prepared radiopharmaceuticals must still address core attributes such as accuracy, precision, specificity, detection and quantification limits, linearity, and range. However, these parameters must be reinterpreted considering the distinct characteristics of radiopharmaceuticals. Due its short half-life, ^68^Ga presents significant challenges related to its decay kinetics, the time-sensitivity of quality control procedures, and the limited availability of reference materials for radiolabelled impurities.

Moreover, an analytical method should undergo re-validation whenever significant changes occur that could impact its reliability or performance. These changes include the following:

- Modifications in the radiopharmaceutical preparation process that may introduce new or different impurities not previously considered, such as a change in purification strategy or the use of an alternative precursor;
- Alterations in the final product composition, including increased radioactivity levels or the introduction of different excipients;
- Substantial adjustments to the analytical procedure itself, for instance, the replacement of the HPLC column with one featuring a different stationary phase, or major changes to the mobile phase composition [69].

Additional regulatory guidelines relevant to radiopharmaceuticals include ICH Q3C (R9) “Residual solvents” [70] and ICH Q3D (R2) “Guideline for Elemental Impurities” [71]. According to ICH Q3C (R9), solvents commonly used in radiosynthesis, such as ethanol and acetic acid, are classified as Class 3 solvents, characterised by low toxic potential. However, their residual content must be carefully monitored. ICH Q3D (R2), on the other hand, establishes permissible daily exposure limits for elemental impurities. For [^68^Ga]GaCl_3_ solutions eluted from ^68^Ge/^68^Ga generators, zinc and iron are recognised as the principal elemental impurities, with their acceptable limits defined in the corresponding European Pharmacopoeia monograph [72]. In the case of cyclotron-produced ^68^GaCl_3_, additional elements such as nickel and copper must also be considered and appropriately controlled to ensure product safety [73].

## 4. Overview of Main ^68^Ga Tracers Extemporaneously Prepared

### 4.1. PSMA Tracers

Prostate-specific membrane antigen (PSMA) is a type II transmembrane glycoprotein predominantly expressed in prostatic tissue, with expression levels significantly increased in prostate cancer cells [74], particularly in high-grade, metastatic, and hormone-refractory tumours [75]. This expression profile makes PSMA an ideal molecular target for both diagnostic and therapeutic applications in prostate cancer.

**Figure 5:**
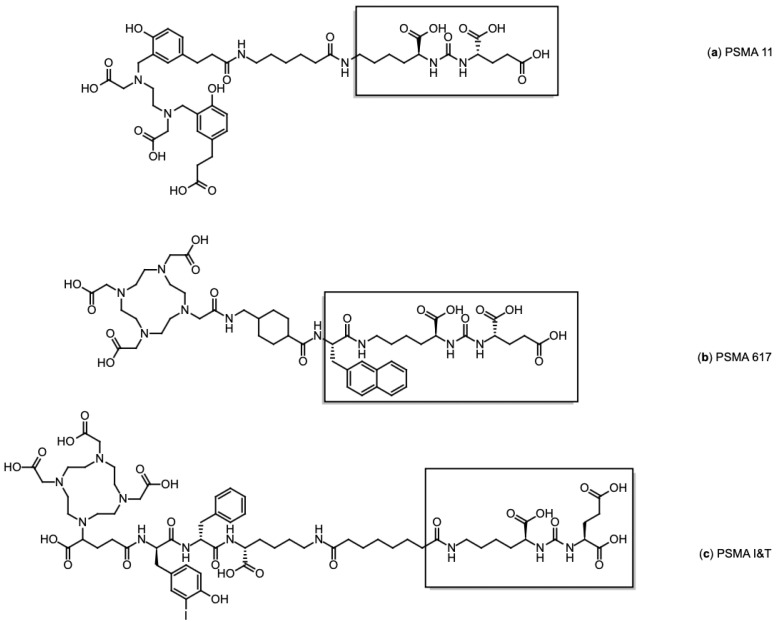
Chemical structures of PSMA ligands: (**a**) PSMA-11, (**b**) PSMA-617, and (**c**) PSMA I&T. The PSMA binding motif is in brackets.

Among the various radiolabelled compounds developed for PSMA-targeted imaging, three low-molecular-weight ligands, PSMA-11, PSMA-617, and PSMA-I&T, have gained prominence. Their chemical structures, along with the conserved PSMA-binding motif, are illustrated in Figure 5.

[^68^Ga]Ga-PSMA-11 (Glu-NH-CO-NH-Lys(Ahx)-[^68^Ga(HBED-CC)]) (Figure 5a), the first ^68^Ga-labelled radiopharmaceutical approved by the FDA for PET imaging in prostate cancer, was developed by the Heidelberg group [76]. Eder et al. demonstrated its high affinity for human PSMA and its efficient internalisation in PSMA-positive tumour cells. Its biodistribution closely mirrors the physiological and pathological expression of PSMA, supporting its clinical use for accurate tumour localisation and staging. Other PSMA-directed radiotracers, including [^68^Ga]Ga-PSMA-617 (Figure 5b) and [^68^Ga]Ga-PSMA-I&T (Figure 5c), exhibit comparable pharmacokinetic and imaging characteristics, further validating the robustness of PSMA as a target for prostate cancer imaging [77]. However, despite their diagnostic capability, these two compounds are primarily employed in therapeutic contexts, as their structural design is compatible with therapeutic radionuclides such as ^177^Lu, making them more suitable for radioligand therapy than for routine PET imaging [78]. Accordingly, PSMA-617 has only been approved by FDA/EMA in its therapeutic form labelled with ^177^Lu, while [^68^Ga]Ga-PSMA-I&T and [^177^Lu]Lu PSMA-I&T remain investigational and are currently limited to clinical studies.

### 4.2. DOTATOC and Somatostatin Receptor Tracers

**Figure 6:**
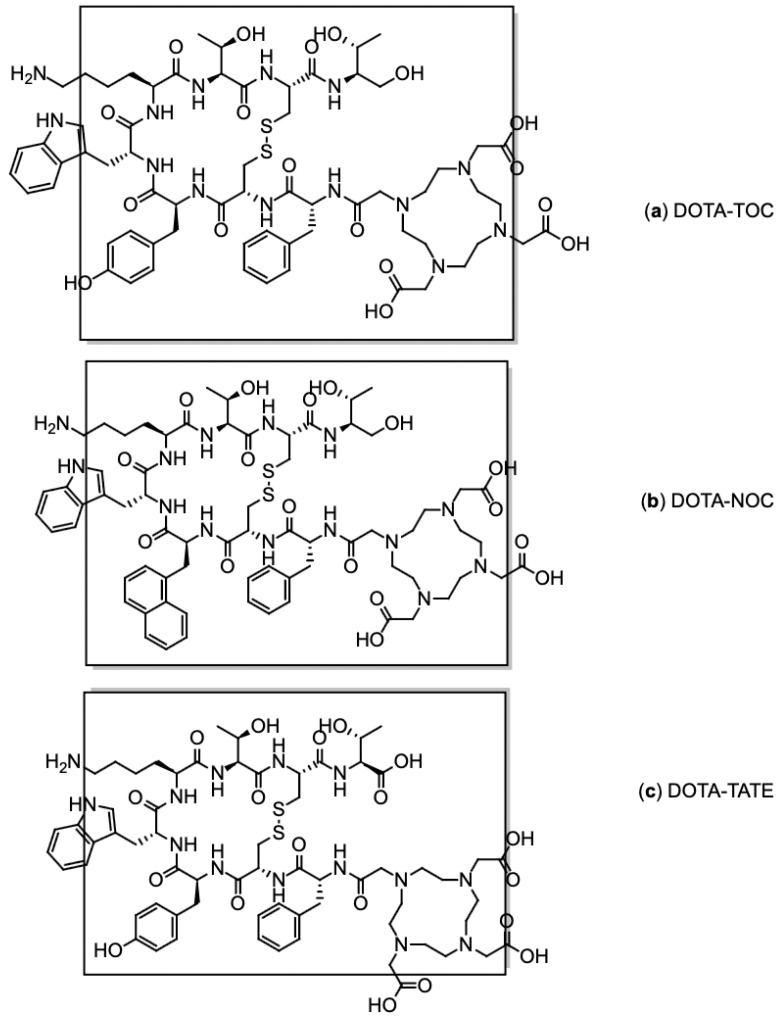
Chemical structures of SSR ligands: (**a**) DOTA-TOC, (**b**) DOTA-NOC, and (**c**) DOTA TATE. The SSR binding motif is in brackets.

Three main types of [^68^Ga]Ga-DOTA-conjugated peptides are routinely employed in clinical practice: [^68^Ga]Ga-DOTA-TOC ([Ga-DOTA-D-Phe^1^-Tyr^3^]octreotide, Figure 6a), [^68^Ga]Ga-DOTA-NOC ([Ga-DOTA-D-Phe^1^-1NaI^3^]octreotide, Figure 6b), and [^68^Ga]Ga-DOTA-TATE ([Ga-DOTA-D-Phe^1^-Tyr^3^]octreotate, Figure 6c). These radiolabelled somatostatin analogues are SSTR agonists, specifically developed to target somatostatin receptors (SSRs), which are overexpressed on the surface of neuroendocrine tumour (NET) cells [79]. They are capable of binding to the receptor and triggering internalisation, a key feature that facilitates intracellular accumulation of the radiotracer and enables both effective imaging and targeted radionuclide therapy. Internalisation into tumour cells is considered a hallmark of the diagnostic and therapeutic efficacy of these compounds [80].

Each compound exhibits high binding affinity for distinct SSR subtypes: Both DOTA-TOC and DOTA-TATE predominantly target SSR2 and show moderate affinity for SSR5 [81], whereas DOTA-NOC also demonstrates binding to SSR3 [82]. Among them, [^68^Ga]Ga-DOTA-TOC is the most widely used in routine clinical practice due to its strong affinity for SSR2 and favourable interaction with SSR5, offering an optimal balance between diagnostic sensitivity and receptor coverage.

### 4.3. FAPI Tracers

While most diagnostic and therapeutic strategies have traditionally focused on targeting tumour cells, increasing attention is now being directed toward the tumour microenvironment (TME). The TME is a complex environment composed of immune cells, vasculature, extracellular matrix components, and cancer-associated fibroblasts (CAFs). Among the limited number of available biomarkers for CAFs, the fibroblast activation protein (FAP) has emerged as one of the most promising. FAP is a membrane-bound serine protease and transmembrane glycoprotein that is selectively overexpressed on activated fibroblasts within the stroma of various solid tumours [83].

**Figure 7:**
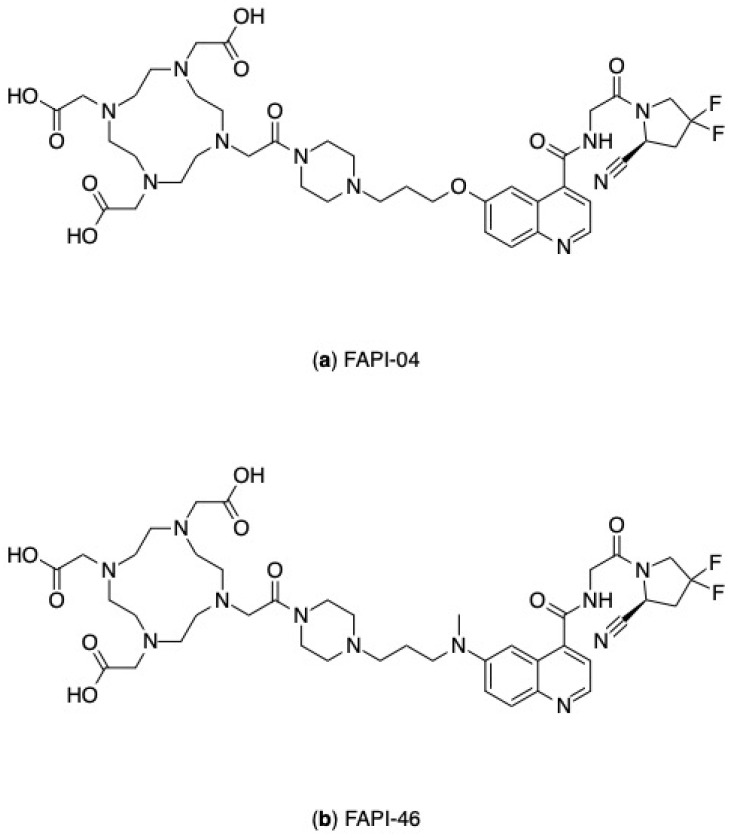
Chemical structures of FAPI compounds: (**a**) FAPI-04, (**b**) FAPI-46.

Preliminary evidence has generated interest in FAP as the next target in the field of nuclear medicine [84]. A range of radiolabelled fibroblast activation protein inhibitors (FAPI) are currently under investigation as PET imaging agents across multiple tumour types, with several compounds also showing potential for theranostic applications. Notable examples include FAPI-04 and FAPI-46, as illustrated in Figure 7.

## 5. Extemporaneous Formulations and Quality Control of ^68^Ga Radiopharmaceuticals

### 5.1. Production of Extemporaneous Formulations of ^68^Ga Radiopharmaceuticals for Clinical Uses

**Figure 8:**
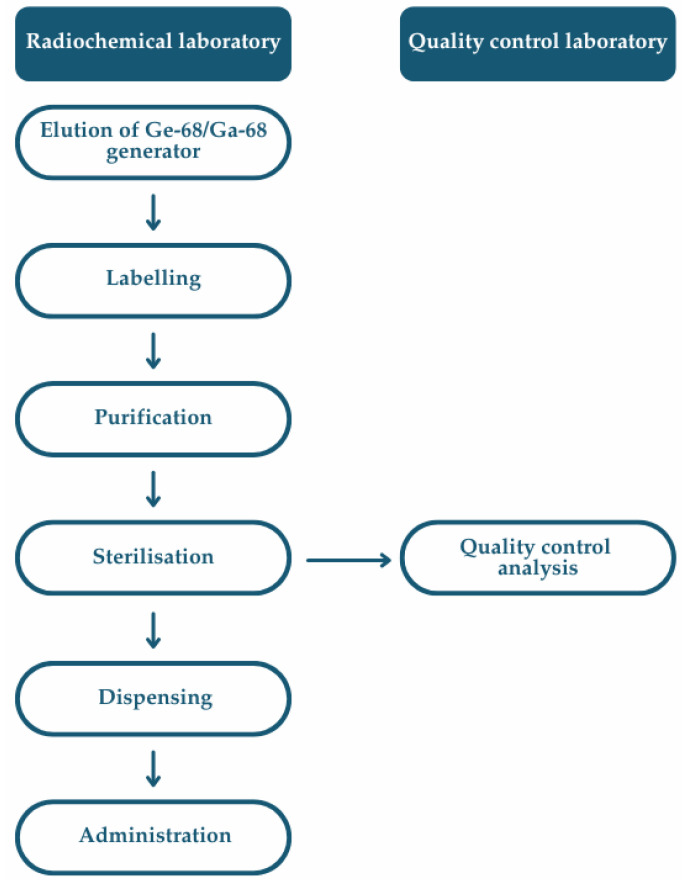
Flowchart of a multistep extemporaneous preparation of ^68^Ga-labelled radiopharmaceuticals, including radiolabelling, purification, formulation, and sterile filtration to yield an injectable product suitable for clinical administration.

According to the Eur. Ph. [85], the preparation of extemporaneous radiopharmaceuticals involves a series of critical operations, including the purchasing of raw materials, the production or elution of the radionuclide, radiolabelling, formulation, purification (where applicable), sterilisation, quality control analysis, dispensing, and final product release to the patients (Figure 8). Each of these steps must be performed within a structured quality management system, guided by principles of risk assessment and aligned with current Good Radiopharmacy Practice (cGRPP) [47], as well as relevant national and European regulatory frameworks.

The process typically begins with the elution of [^68^Ga]GaCl_3_ from a ^68^Ge/^68^Ga generator or via cyclotron-based production, as described in Section 2.2 and according to the Eur. Ph. monograph 2464 [86]. Manual radiolabelling synthesis is performed within a Grade A laminar flow isolator, whereas automated synthesis is typically carried out inside a shielded Grade C hotcell. Following sterilisation, the dispensing of the final product takes place within a shielded Grade A environment to ensure sterility [87]. Overall, these activities are carried out within a Grade C or D cleanroom, depending on the sterility requirements and the complexity of the radiopharmaceutical preparation process.

Ensuring the sterility of radiopharmaceutical preparations is a fundamental prerequisite for patient safety. In the case of ^68^Ga-labelled radiopharmaceuticals, achieving sterility poses particular challenges due to the physicochemical properties of the compounds and the short half-life of the radionuclide, which necessitate rapid preparation, quality control, and release within tight time constraints. Terminal sterilisation by autoclaving is considered the gold standard for sterile drug products; however, it typically involves exposure to steam at 121 °C for at least 15 min and, therefore, this method is not feasible for ^68^Ga-radiopharmaceuticals. These formulations are highly sensitive to heat; elevated temperatures can induce molecular degradation, loss of radiochemical purity, and the formation of impurities. Additionally, the time associated with autoclaving would result in significant radioactive decay, thereby compromising the clinical utility of the product [88,89]. For this reason, sterilisation is typically achieved through sterile filtration, unless the nature of the compound precludes it, in which case strict aseptic techniques become critical. Consequently, sterile filtration followed by aseptic handling is the preferred approach to ensure both the microbiological safety and the preservation of the chemical and radiochemical integrity of ^68^Ga-radiopharmaceuticals. In European radiopharmacy practice, sterile filtration is widely implemented as a standard procedure, in compliance with Eur. Ph. and national regulations [90]. When properly integrated into an aseptic workflow and supported by validated integrity testing, it ensures microbiological safety even in time-sensitive, small-scale extemporaneous preparations. However, the selection of the filter membrane is a critical factor, as certain materials may interact with the radiopharmaceutical compound, potentially leading to reduced radiochemical yield. Therefore, compatibility studies are essential to ensure that the filter does not adsorb the radiolabelled product or alter its chemical integrity.

#### 5.1.1. Manual Production

Radiolabelling with ^68^Ga can be carried out manually through a simple process. Initially, the reaction mixture is prepared by combining [^68^Ga]GaCl_3_ with a suitable buffer, the precursor molecule, and any required additives. The solution is then incubated for a defined period under specific temperature conditions to enable efficient complexation of the radiometal. Following incubation, purification may be performed using a solid-phase extraction (SPE) cartridge. The purified product is then eluted and passed through a sterile filter, yielding the final injectable formulation [62].

This method can be adapted to accommodate different precursors and formulation needs. A practical example of this approach is implemented at the Nuclear Medicine Department of the Policlinico di Bari, where [^68^Ga]Ga-PSMA-11 is routinely prepared. In this protocol, 5 mL of [^68^Ga]GaCl_3_ in 0.1 N HCl is incubated at room temperature for 7.5 min with 60 mg of acetic acid buffer and 20 µg of PSMA-11 precursor. Upon completion of the radiolabelling reaction, the solution is reformulated by adding 5 mL of 0.9% NaCl, then it is filtered through a 0.22 µm sterile filter to obtain the final product, ready for administration. Radiochemical purity consistently exceeds 99% as assessed by HPLC, and the overall radiolabelling yield approaches 100% decay corrected.

#### 5.1.2. Automated Production

**Figure 9:**
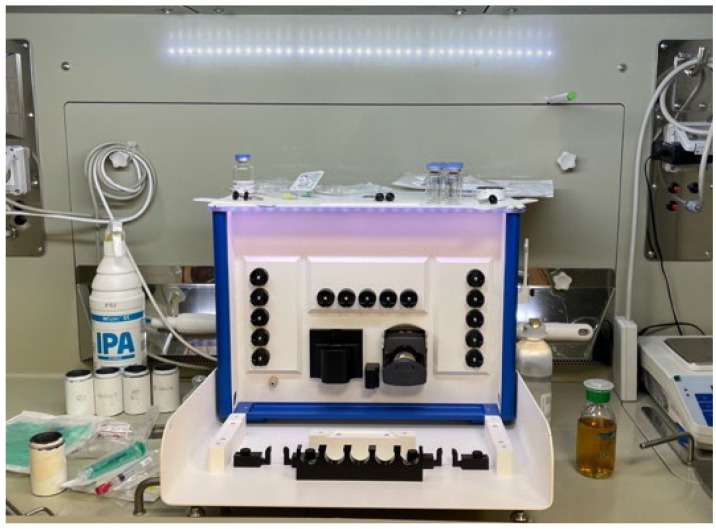
Example of a synthesis module for the preparation of ^68^Ga radiopharmaceuticals, installed within a shielded hot cell in a hospital radiopharmacy. The module is equipped with pre-defined positions for reagents, cassettes, and waste containers, enabling standardised and operator-independent execution of radiolabelling procedures under aseptic and radiologically safe conditions.

The increasing clinical demand for ^68^Ga radiopharmaceuticals has accelerated the adoption of automated synthesis platforms, which provide clear advantages in terms of process reproducibility, radiochemical yield, operator safety, and compliance with GMP standards [91]. Most commercially available systems in use employ cassette-based technology (Figure 9). In these platforms, single-use disposable cassettes are replaced after each synthesis run, ensuring sterility, minimising cross-contamination, and facilitating batch-to-batch standardisation [62]. The use of disposable, pre-sterilised cassettes is considered best practice to reduce microbial risk and radiation exposure, while also simplifying cleaning procedures and minimising operator intervention [68].

**Figure 10:**
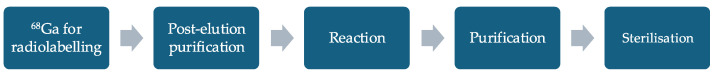
Schematic overview of the general workflow for the preparation of ^68^Ga-labelled radiopharmaceuticals.

Similar to manual procedures, automated ^68^Ga-labelling processes comprises four key steps: (1) production of ^68^Ga from a generator or a cyclotron; (2) SPE purification to concentrate the eluate and remove metallic or ionic impurities; (3) preparation of the reaction mixture containing the precursor, buffer, and optional additives; and (4) final purification of the labelled product prior to sterile filtration and formulation for clinical use (Figure 10).

A common challenge in automated workflows is the relatively large elution volume (5–10 mL), which may require post-[^68^Ga]GaCl_3_ production processing to concentrate the activity and remove metallic or ionic impurities prior to the radiolabelling step. Three purification strategies are commonly employed:

- Fractionation, which isolates the most active portion of the eluate, although this approach may lead to activity losses and requires higher precursor amounts [79];
- Anion-exchange chromatography, which traps the anionic [GaCl_4_]^−^ complex formed at HCl concentrations > 5.5 M and releases Ga^3+^ upon elution with water [34];
- Cation-exchange chromatography, which retains Ga^3+^ while removing metal impurities (e.g., ^68^Ge, Zn^2+^, Fe^3+^) using acetone/HCl or alternative eluents such as HCl/NaCl. As acetone is not suitable for injection, its presence must be quantified by gas chromatography prior to clinical use [62].

Following purification, the [^68^Ga]GaCl_3_ solution is introduced into a reaction vessel containing the reaction mixture: buffer, precursor, and any required additives. After the radiolabelling step, a purification procedure may be performed if required. In most cases, reversed-phase C18 cartridges are employed to retain the labelled product. Unreacted ^68^Ga species, typically in ionic form, do not bind to the cartridge and are flushed into the waste stream. The radiolabelled compound, along with any colloidal forms of ^68^Ga, is retained on the stationary phase. Subsequently, the final product is eluted using an ethanol/water mixture, passed through a 0.22 µm sterile filter, and collected in the final product vial. Colloidal gallium species remain adsorbed on the C18 phase, thereby improving the radiochemical purity of the final formulation.

Several examples illustrate the versatility of automated production:

- Fuscaldi et al. [92] described the synthesis of [^68^Ga]Ga-PSMA-11 using the Modular-Lab PharmTracer module. In this protocol, PSMA-11 (10–20 µg) is dissolved in 1.0 mL of 0.1 M sodium acetate buffer (pH 4.5). ^68^Ga is eluted from a ^68^Ge/^68^Ga generator with 0.1 M HCl, purified via cation exchange, and transferred into the reaction vial. Labelling occurs at 85 °C for 3–5 min, followed by C18 cartridge purification and sterile filtration, completing the process in approximately 25 min. The final product exhibits >99% radiochemical purity and is clinically suitable.
- Fouillet et al. [93] developed a method transposable to PSMA I&T and PSMA-617 using the GAIA^®^ synthesis module (Elysia-Raytest, GmbH, Straubenhardt, Germany). Here, 10 µg of PSMA-11 is dissolved in 2.8 mL of 0.08 M ammonium acetate buffer (pH 4.6), with ^68^Ga eluted and concentrated via SCX cartridge, then labelled at 97 °C for 8 min. Purification through a C18 cartridge and sterile filtration yields a product meeting GMP clinical-grade standards with >91% radiochemical purity within 27 min.
- The Nuclear Medicine Department of Policlinico di Bari routinely synthesises [^68^Ga]Ga-DOTATOC using the GAIA^®^ synthesis module. In this method, 5 mL of [^68^Ga]GaCl_3_ is labelled with 50 µg of DOTATOC at 90 °C for 10 min. The formulation uses a buffer system of acetic acid, ammonium acetate, and HCl, followed by C18 purification and sterile filtration to obtain the injectable product.
- Spreckelmeyer et al. [94] described the semi-automated production of [^68^Ga]Ga-FAPI-46 using the Modular Lab PharmTracer and Modular Lab eazy systems. After SCX purification and radiolabelling at 95–98 °C for 10 min, the product is purified with a CM cartridge and sterile-filtered. As the CM cartridge traps unreacted ^68^Ga species and flushes [^68^Ga]Ga-FAPI-46 directly in the product vial, this method avoids the use of ethanol, thereby eliminating the need for residual solvent testing.

A comparative overview of different manual and automated ^68^Ga-radiopharmaceutical production methods is shown in Table 2.

#### 5.1.3. Cold Kit

Similarly to the long-established use of ^99m^Tc cold kits in SPECT imaging, cold kit formulations for ^68^Ga-radiolabelling have become a practical and efficient solution for PET applications. These kits typically contain a lyophilised precursor along with excipients such as buffering agents and stabilisers, facilitating the streamlined preparation of ^68^Ga-labelled radiopharmaceuticals in hospital. Among the commercially available options are kits targeting PSMA (e.g., Locametz^®^ by Novartis (Novartis AG, Basel, Switzerland), Illumet™ or Illuccix^®^ by Telix Pharmaceuticals (Telix Pharmaceuticals Ltd., Melbourne, VIC, Australia) and Isoprotrace^®^ by Isotopia (Isotopia Molecular Imaging Ltd., Ramat Gan, Israel), all containing PSMA-11, and GalliProst^®^ by ROTOP (ROTOP Pharmaka GmbH, Dresden, Germany), containing THP-PSMA), somatostatin receptors (e.g., DOTA-TATE, marketed as Netspot^®^ by Novartis), and DOTA-TOC (Somakit-TOC^®^ by Novartis) [18].

An overview of current kit-based formulations is provided by Satpati et al. [95]. For instance, Somakit-TOC^®^ contains 40 µg of DOTA-TOC and is designed to be radiolabelled with 5 mL of ^68^Ga eluate (0.1 N HCl), supporting a maximum activity of up to 1100 MBq. Similarly, GalliProst^®^ includes 40 µg of THP-PSMA along with sodium bicarbonate, mannitol, and phosphate buffer, providing a mixture suitable for rapid complexation under mild conditions [96].

### 5.2. Quality Control Analysis

According to current guidelines, quality control of ^68^Ga extemporaneous formulations of radiopharmaceuticals must include the following tests [59,90]:

- Appearance: Radiopharmaceuticals must be clear, colourless solutions, free of visible particulate matter or turbidity. Visual inspection is typically performed under a calibrated light source using a clean glass inspection station or white background panel.
- pH: The final formulation, intended for intravenous administration, should exhibit a pH compatible with parenteral use, typically within the range of 3.5 to 8.5. For generator eluates, the pH is expected to be below 2 to prevent the precipitation of gallium hydroxide. Measurements are performed using benchtop pH meters with microelectrodes.
- Radionuclidic identity: established via half-life measurement (acceptable range: 62–74 min) and γ-emission spectrum, with characteristic peaks at 511 and 1077 keV. This requires a γ spectrometry system with energy-calibrated sodium iodide scintillation crystals or high-purity Germanium detectors.
- Radionuclidic purity: The level of long-lived impurities, particularly ^68^Ge breakthrough, must be below 0.001% of total radioactivity. Additional limits are set for any other unintended radionuclidic species. γ spectroscopy with shielding and spectral analysis software is used to quantify breakthrough and confirm compliance. Since ^68^Ge has a much longer half-life than ^68^Ga, this analysis must be performed after product release, once the sample has decayed sufficiently, in order to avoid interference from ^68^Ga and allow accurate quantification of long-lived contaminants.
- Radiochemical purity: Defined as the percentage of radioactivity bound to the intended compound, radiochemical purity must exceed 95%, as unbound ^68^Ga (e.g., free Ga^3+^ or colloidal species) could compromise diagnostic quality. Analytical procedures include thin-layer chromatography (iTLC) and high-performance liquid chromatography (HPLC), both equipped with radiometric detectors.
- Residual solvents: If organic solvents are used during synthesis (e.g., ethanol, acetic acid), their presence in the final formulation must be quantified by gas chromatography equipped at least with a flame ionisation detector (GC-FID), typically with headspace injection to comply with ICH guidelines.
- Bacterial endotoxins: evaluated using the Limulus Amebocyte Lysate (LAL) assay. Endotoxin quantification is commonly performed with portable or benchtop systems using kinetic chromogenic or turbidimetric methods (e.g., Endosafe^®^ or similar), with an acceptance limit of ≤175 EU per total volume of the radiopharmaceutical.
- Sterility: While sterility testing is performed retrospectively in accordance to Ph. Eur. 2.6.1), integrity testing of the sterile filter (e.g., bubble point test) must be completed prior to batch release using validated integrity testers; sterility testing and sterile filtration are conducted in Grade A environments with Grade B background, typically inside shielded isolators or laminar flow hot cells.
- Radioactivity content and concentration: measured using ionisation chamber-based dose calibrators routinely cross-calibrated with secondary standards to ensure correct activity per administered volume.
- Specific or molar activity: Although not always required, the (apparent) specific activity (e.g., MBq/µg) or molar activity (e.g., MBq/nmol) can be reported, acknowledging the presence of excess unlabelled precursor or ligand in most preparations.

These extensive controls are not necessary when radiopharmaceuticals are prepared using a kit with a marketing authorisation. In this case, pre-release quality control analyses are detailed in the Summary of Product Characteristics (SmPC) of the marketing authorisation. These typically encompass several standardised assessments:

- Visual inspection for particulate matter and colour changes;
- pH determination using either a pH meter or indicator strips;
- Radiolabelling efficiency evaluation through iTLC;
- Radioactivity measurement using a dose calibrator.

An illustrative example of such standardised quality control protocols is found in the Locametz^®^ kit preparation procedures.

## 6. Conclusions

The extemporaneous preparation of ^68^Ga-labelled radiopharmaceuticals plays a key role in expanding access to personalised diagnostic agents in nuclear medicine. This flexible and efficient approach allows for the rapid, on-demand production of radiopharmaceuticals tailored to specific clinical indications, ensuring timely patient management and optimising the use of short-lived radionuclides such as ^68^Ga [97].

As the field continues to evolve, one of the most promising future directions is extending extemporaneous preparation beyond diagnostic applications to include therapeutic radiopharmaceuticals. The increasing interest in theranostic approaches and the development of novel therapeutic radionuclides suggest the feasibility of preparing radiopharmaceutical therapies directly in-house [98]. This would represent a significant step forward in the personalisation of nuclear medicine, enabling truly patient-specific treatment strategies to be implemented rapidly and efficiently.

The continued advancement of this practice will depend on the refinement of synthesis protocols, the development of reliable automated systems, and the strengthening of multidisciplinary collaborations. Extemporaneous preparations are not only a practical solution for today’s diagnostic challenges, but they also represent a strategic framework toward the future of individualised radiopharmaceutical therapy.

## Figures and Tables

**Table 1:** Comparison of the physical characteristics of clinically relevant Ga radioisotopes, including half-life, maximum energy, and principal type of radiation emitted.

Radionuclide	Half-Life	E_max_ (keV)	Radiation
^66^Ga	9.5 h	4153	β^+^ (56%)
^67^Ga	78.3 min	91, 93, 185, 296, 388	γ
^68^Ga	67.7 min	1899, 770	β^+^ (89%)

**Table 2:** Summary of ^68^Ga-radiopharmaceutical production methods.

Method	Precursor	Reaction Buffer	Incubation	Purification	Final Processing	Time (min)
Manual [^68^Ga]Ga-PSMA-11 (Policlinico Bari)	20 µg PSMA-11	Sodium acetate buffer	7.5 min, RT	None	NaCl addition + Filtration	~15
Automated [^68^Ga]Ga-PSMA-11 (Fuscaldi et al. [92])	10–20 µg PSMA-11	0.1 M Sodium acetate	85 °C, 3–5 min	C18 Cartridge	Sterile Filtration	~25
Automated [^68^Ga]Ga-PSMA-11 (Fouillet et al. [93])	10 µg PSMA-11	0.08 M Ammonium acetate	97 °C, 8 min	C18 Cartridge	Sterile Filtration	~27
Automated [^68^Ga]Ga-DOTATOC (Policlinico Bari)	50 µg DOTATOC	Acetic acid, Ammonium acetate, HCl	90 °C, 10 min	C18 Cartridge	Sterile Filtration	~30
Semi-Automated [^68^Ga]Ga-FAPI-46 (Spreckelmeyer et al. [94])	50 µg FAPI-46	Acetate/Sodium ascorbate	95–98 °C, 10 min	CM Cartridge	Sterile Filtration	~30
